# Loss of SETD2 aggravates colorectal cancer progression caused by SMAD4 deletion through the RAS/ERK signalling pathway

**DOI:** 10.1002/ctm2.1475

**Published:** 2023-11-14

**Authors:** Chunxiao Ma, Min Liu, Wenxin Feng, Hanyu Rao, Wei Zhang, Changwei Liu, Yue Xu, Ziyi Wang, Yan Teng, Xiao Yang, Li Ni, Jin Xu, Wei‐Qiang Gao, Bing Lu, Li Li

**Affiliations:** ^1^ State Key Laboratory of Systems Medicine for Cancer Renji‐Med X Clinical Stem Cell Research Center, Ren Ji Hospital, School of Medicine and School of Biomedical Engineering, Shanghai Jiao Tong University Shanghai China; ^2^ School of Biomedical Engineering and Med‐X Research Institute, Shanghai Jiao Tong University Shanghai China; ^3^ State Key Laboratory of Proteomics, Beijing Proteome Research Center, National Center for Protein Sciences Beijing Institute of Lifeomics Beijing China; ^4^ Department of Nursing Shanghai East Hospital, Tongji University Shanghai China; ^5^ Department of General Surgery, Department of Colorectal Surgery, Shanghai East Hospital School of Medicine, Tongji University Shanghai China

**Keywords:** CRC, DUSP7, RAS/ERK signalling pathway, SETD2, SMAD4

## Abstract

**Backgound:**

Colorectal cancer (CRC) is a complex, multistep disease that arises from the interplay genetic mutations and epigenetic alterations. The histone H3K36 trimethyltransferase SET domain‐containing 2 (SETD2), as an epigenetic signalling molecule, has a 5% mutation rate in CRC. SETD2 expression is decreased in the development of human CRC and mice treated with Azoxymethane /Dextran sodium sulfate (AOM/DSS). Loss of SETD2 promoted CRC development. SMAD Family member 4 (SMAD4) has a 14% mutation rate in CRC, and SMAD4 ablation leads to CRC. The co‐mutation of SETD2 and SMAD4 predicted advanced CRC. However, little is known on the potential synergistic effect of SETD2 and SMAD4.

**Methods:**

CRC tissues from mice and SW620 cells were used as research subjects. Clinical databases of CRC patients were analyzed to investigate the association between SETD2 and SMAD4. SETD2 and SMAD4 double‐knockout mice were established to further investigate the role of SETD2 in SMAD4‐deficient CRC. The intestinal epithelial cells (IECs) were isolated for RNA sequencing and chromatin immunoprecipitation sequencing (ChIP‐seq) to explore the mechanism and the key molecules resulting in CRC. Molecular and cellular experiments were conducted to analyze the role of SETD2 in SMAD4‐deficient CRC. Finally, rescue experiments were performed to confirm the molecular mechanism of SETD2 in the development of SMAD4‐dificient CRC.

**Results:**

The deletion of SETD2 promotes the malignant progression of SMAD4‐deficient CRC. *Smad4^Vil‐KO^; Setd2^Vil‐KO^* mice developed a more severe CRC phenotype after AOM/DSS induction, with a larger tumour size and a more vigorous epithelial proliferation rate. Further mechanistic findings revealed that the loss of SETD2 resulted in the down‐regulation of DUSP7, which is involved in the inhibition of the RAS/ERK signalling pathway. Finally, the ERK1/2 inhibitor SCH772984 significantly attenuated the progression of CRC in *Smad4^Vil‐KO^;Setd2^Vil‐KO^* mice, and overexpression of DUSP7 significantly inhibited the proliferation rates of SETD2^KO^; SMAD4^KO^ SW620 cells.

**Conclusions:**

Our results demonstrated that SETD2 inhibits the RAS/ERK signaling pathway by facilitating the transcription of DUSP7 in SMAD4‐deficient CRC, which could provide a potential therapeutic target for the treatment of advanced CRC.

## Graphical abstract

- SETD2 deficiency promotes the progression of SMAD4‐deficient CRC.
- SETD2 deletion exacerbates SMAD4‐deficient CRC via RAS/ERK signalling pathway.
- SETD2 inhibits the RAS/ERK signalling pathway by promoting DUSP7 transcription.

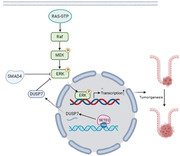

## 1 INTRODUCTION

The development of colorectal cancer (CRC) is a highly intricate and multistep process that results from the interplay between genetic mutations and epigenetic modifications. The molecular pathogenesis of this disease involves dysregulation of numerous cellular pathways, including cell proliferation, apoptosis, differentiation and DNA repair. In China, CRC is the third most common cancer. The survival rates are significantly reduced in the middle and advanced stages of CRC. There are the two main factors in the development of CRC, gene mutation and epigenetic regulation instability.^1^, ^2^ In the middle and advanced stages of CRC, the survival rates are significantly reduced. Typically, CRC arises from dysplasia of the epithelial crypts. The steps involved in the transition from normal epithelium to adenocarcinoma were described in the 1990 model of cancer progression.^3^ Currently, it has been pointed out that gene mutation and epigenetic regulation instability are the two main causes of the development of CRC.^3^, ^4^, ^5^

SMAD Family Member 4 (SMAD4) is the core signalling transduction molecule in the transforming growth factor‐β (TGF‐β) signalling pathway. Loss of SMAD4 occurs in about 30% in CRC cases,^6^ and its deficiency increases the risk of recurrence and poor prognosis of CRC patients. SMAD4 has two major structural domains, MH1, which binds to DNA, and MH2, which interacts with MH1 for transcriptional regulatory activity.^7^ It has been reported that the TGF‐β pathway plays an oncogenic role in CRC.^7^, ^8^ As an important intracellular receptor signalling molecule in TGF‐β signalling pathway, SMAD4 can enter the nucleus by forming homologous complexes alone or with other activated SMAD family members. At present, many studies have reported that the loss of SMAD4 can cause gastrointestinal cancer.^9^, ^10^, ^11^, ^12^

Histone modification is one of the important epigenetic regulation forms. SET domain‐containing 2 (SETD2) is the only known H3K36 trimethyltransferase,^13^, ^14^ and frequently mutated (5%) in CRC samples.^15^ The mutation rate of SETD2 is as high as 17% in UC. SETD2‐mediated trimethylation of H3K36 is involved in a range of cellular processes, including DNA damage repair, transcriptional regulation and selective splicing.^16^ SETD2 comprises several structural domains, of which the SET structural domain exerts protein methyltransferase activity.^17^ It has been reported that SETD2 plays a suppressor role in tumours, such as renal cancer, gastric cancer, colon cancer and pancreatic cancer.^14^ SETD2 inhibited the WNT signalling pathway and suppressed small intestinal tumour formation by regulating the variable spliceosome to inhibit DVL2 upstream of the WNT pathway.^18^ Loss of SETD2 contributes to the development of aggressive gastrointestinal stromal tumours.^19^ SETD2 inhibits intestinal epithelial injury by regulating oxidative stress‐related factors.^15^ SETD2 also plays a role in inhibiting epithelial healing.^20^ In addition, SETD2 has been studied in many developmental areas regarding sperm development, V(D)J recombination, bone marrow mesenchymal stem cell differentiation, occurrence and poor prognosis of MDS‐related leukaemia, regulation of hepatic lipid metabolism and its role in embryonic development.^21^, ^22^, ^23^, ^24^, ^25^, ^26^ However, the role of H3K36 trimethyltransferase SETD2 in TGF‐β signalling deficiency‐induced CRC remains unknown.

Interestingly, loss of SMAD4 occurs in about 30% in CRC cases, and SETD2 has a 5% mutation rate in CRC. These data indicate that SETD2 and SMAD4 play important roles in CRC. It is worth noting that the co‐mutation of SETD2 and SMAD4 predicted advanced CRC in The Cancer Genome Atlas (TCGA) database, and little is known on the potential synergistic effect of SETD2 and SMAD4. To explore whether SETD2 deficiency promotes the advanced CRC in absence of SMAD4, mice models with intestinal epithelial cell (IEC)‐specific SETD2 and SMAD4 mutations were generated and we found that SETD2 deficiency exacerbates SMAD4‐mutant CRC. Mechanistically, our findings highlighted that SETD2 was a critical epigenetic regulator in the SMAD4‐deficient CRC through RAS/ERK signalling pathway.

## 2 MATERIALS AND METHODS

### 2.1 Mice strains

All the mice were born and maintained in the specific pathogen‐free animal facility and fed with C57BL/6j as the background. The Setd2‐flox mice were generated by Shanghai Biomodel Organism Co., and Villin‐Cre mice were purchased from Shanghai Bomade Biotechnology Co. Ltd. The Smad4‐flox mice were gifted by Dr. Xiao Yang from Beijing Institute of Lifeomics. These mice were crossed in various combinations to generate models used in this study. In the experiment, their littermates with the same treatment were used as the control. Mice were given intraperitoneal injections with azoxymethane (AOM) (10 mg/kg; Sigma) at 8 weeks of age. After 5 days, the injected mice were fed drinking water with 2% dextran sodium sulphate (DSS) (MP Biomedicals) for 5 days, then the drinking water without DSS was followed for 14 days.^15^, ^27^ After three rounds of DSS treatment, the mice were sacrificed after weight loss to 80% of original body weight or 90 days of AOM injection. For SCH772984 (12.5 mg/kg, MCE) treatments, mice were given intraperitoneal injections twice a day for 14 days after the end of the first cycle of DSS. All experimental procedures were approved by the Institutional Animal Care and Use Committee of Shanghai Jiao Tong University. The ethical number of animal experiments is 2021019.

### 2.2 Isolation of IECs

IECs were isolated from mice colons. The colon tissue obtained from mice were cut into small fragments of about 1 mm and incubated at 37°C for 15 min with 8 mM EDTA. After 15 min, replace the EDTA with PBS and shake vigorously for 45 s. The procedure was repeated, and the supernatant obtained twice was combined. After filtration with a 100‐μm cell sieve, the supernatant was centrifuged at 2000 rpm/min at 4°C for 2 min. Pour out the supernatant and use cold PBS to resuscitate.

### 2.3 Histology, haematoxylin–eosin staining and immunohistochemistry

After washing colon tissues with cold PBS, they were rolled up like a Swiss roll and fixed in 4% paraformaldehyde at 4°C overnight. Intestinal tissues were dehydrated and processed and encapsulated in paraffin. Haematoxylin–eosin (H&E) staining was performed using 5 μm sections. For immunohistochemistry (IHC) staining, the sections were deparaffinised with alcohol, antigen repair was performed in citrate buffer for 4 min on high heat and 12 min on low heat. Endogenous peroxidase was quenched with 3% H_2_O_2_. Next, samples were blocked with 10% BSA. The primary antibody was dropped onto the samples and incubated. The primary antibodies were anti‐SMAD4 (#46535; CST), anti‐CDX2 (ab76541; Abcam), anti‐p‐p44/p42 MAPK (#4370; CST), anti‐Ki67 (ab15580; Abcam) and anti‐DUSP7 (26910‐1‐AP; Proteintech). DAB was used after incubating with secondary antibodies, and then counterstained with haematoxylin and sealed using neutral gum.

### 2.4 Immunofluorescence

The sections were deparaffinised and rehydrated before antigen repair and cooled to room temperature. Membrane permeabilisation was performed using 0.5% TritonX‐100, followed by PBS washing and blocking with 10% BSA. Then, the primary antibody against SETD2 (A3194; Abclonal) was dropped onto the samples and incubated at 4°C overnight. DAPI was used for nuclear counterstaining after incubating by secondary antibody.

### 2.5 Western blot analysis

Intestinal tissue or IECs proteins were performed as previous described.^15^, ^20^ 12.5 and 6% SDS‐PAGE gels were used to separate the proteins. The primary antibodies were anti‐SMAD4 (#46535; CST), anti‐SETD2 (LS‐C332416), anti‐H3 (ab10799; Abcam), anti‐H3K36me3 (ab9050; Abcam), anti‐AKT (#2920; CST), anti‐p‐AKT (#4060; CST), anti‐p44/p42 MAPK (#4370; CST), anti‐p‐p44/p42 MAPK (#4370; CST), anti‐DUSP7 (26910‐1‐AP; Proteintech) and anti‐β‐tubulin (AC008; ABclonal). After incubating with secondary antibodies, the PVDF membranes were washed by TBST. The images were analysed by ImageJ software.

### 2.6 Cell culture, plasmids and transfections

The Human Colorectal Adenocarcinoma Cells SW620 cultured in DMEM containing 1% penicillin–streptomycin and 10% foetal bovine serum at 37°C in a 5% CO_2_ atmosphere. Single‐guide RNA (sgRNA) sequence targeted to SMAD4 was designed,^28^ as well as SETD2.^23^ The sgRNA oligomers were ligated into the LentiCRISPRv2 vector, and lentiviral particles were generated as previous described.^20^ Stable transfection was obtained by infecting cells with viral particles and screening with 2 μg/mL puromycin for 1 week. Human DUSP7 pCMV‐Entry plasmid was purchased from Hunan Fenghui Biotechnology Co., Ltd. Plasmids were transfected in cells using EZ transfection reagent. The SETD2^KO^; SMAD4^KO^ cells were treated with ERK inhibitor SCH772984 at concentration of 300 nmol/L, respectively.

### 2.7 RNA isolation and quantitative RT‐PCR

RNA was extracted from colon IECs using an RNA extraction kit (BioTeke). The cDNA was reverse transcribed with a reverse transcription kit (Takara). β‐actin was used for standardisation. Primers used for RT‐qPCR analysis are listed in Table S1.

### 2.8 | RNA‐seq and analyses

IECs RNA were obtained from *Smad4^Vil‐KO^* and *Smad4^Vil‐K^;Setd2^Vil‐KO^* mice treated with AOM/DSS for 10 days using EDTA‐based isolation. GO analysis and Kyoto encyclopaedia of genes and genomes (KEGG) pathway analysis were used for all differentially expressed genes.

### 2.9 Chromatin immunoprecipitation assay

Chromatin immunoprecipitation and next‐generation sequencing (ChIP‐seq) assay was performed as previous described.^15^ The cells we used were IECs obtained from *Smad4^Vil‐KO^* mice. ChIP‐PCR followed the same steps as ChIP‐seq before quenched with glycine. After lysis of cells with SDS lysate containing protease inhibitors, DNA was broken by sonication to small fragments of 200–1000 bp in size. Antibodies were added and incubated overnight. Protein G Agarose was added to the centrifuge tube incubated overnight for adsorption, beads were washed and elution was subsequently added to elution buffer. Finally, the DNA was released by uncross‐linking and purified.

### 2.10 Data mining using public database

The data of gene expression levels of colon adenocarcinoma/rectum adenocarcinoma oesophageal carcinoma (COAD/READ) (CRC) were obtained from TCGA. The relationship about expression level of two genes were analysed in http://gepia.cancer‐pku.cn.

### 2.11 Statistical analysis

All the data were expressed as mean ± standard deviation (mean ± S.D.). All experiments were repeated at least three times. GraphPad Prism 9.0.0 software was used to analyse all data. Two‐way ANOVA or Student's *t*‐test were used to analyse data. *p* Values < .05 were considered statistically significant. *, *p* < .05; **, *p* < .01; ***, *p* < .001; and ****, *p* < .0001.

## 3 RESULTS

### 3.1 The co‐occurrence rate of SETD2 and SMAD4 mutations is elevated in advanced stage CRC

**FIGURE 1:**
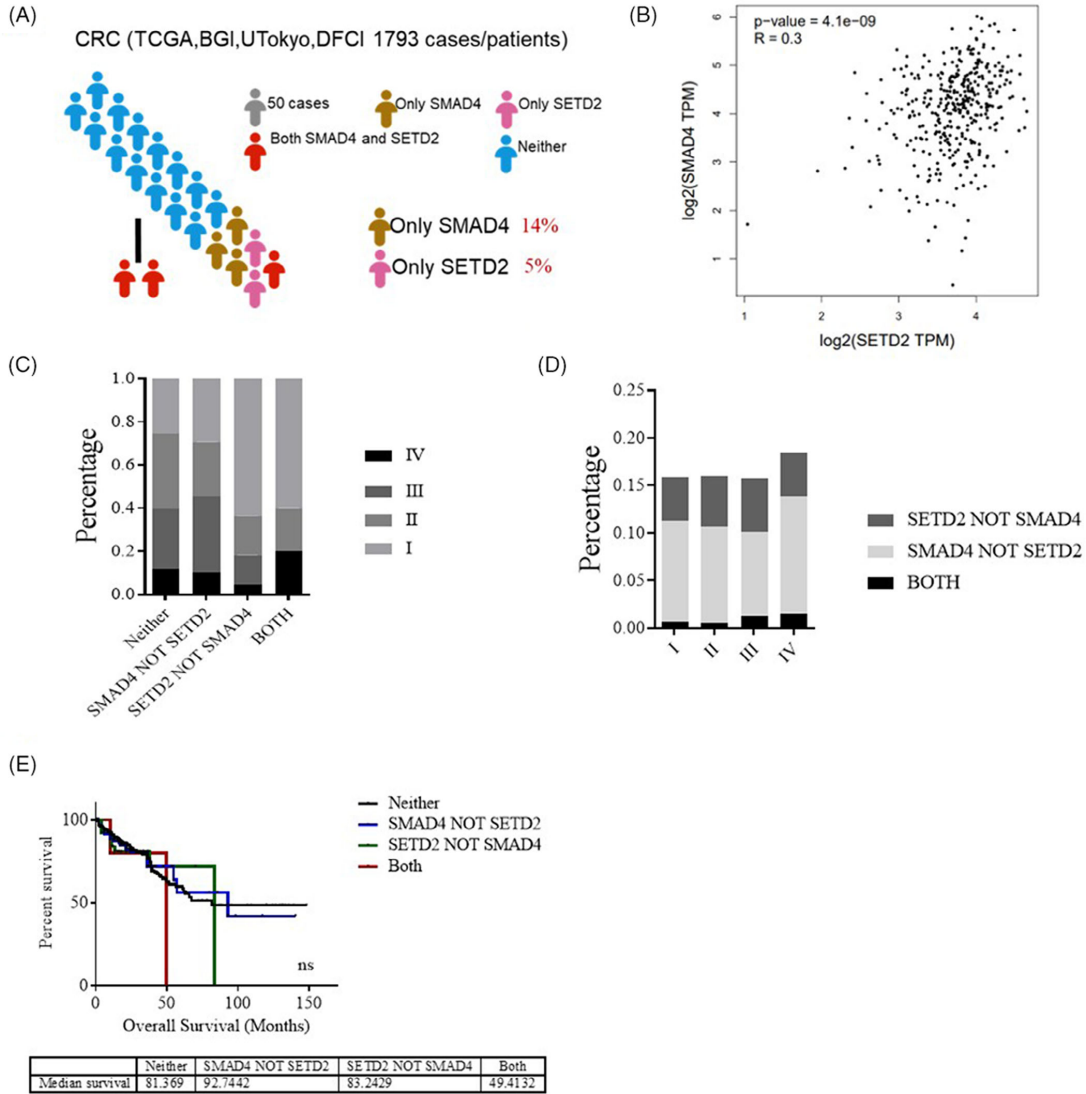
The co‐occurrence rate of SETD2 and SMAD4 mutations are elevated in patients with advanced CRC. (A) Proportion analysis of SMAD4 and SETD2 in 1793 patient samples in TCGA database. (B) Correlation between SMAD4 and SETD2 mRNA expression levels in CRC patients in TCGA database. (C) Proportion analysis between SETD2 and SMAD4 mutations and colorectal cancer stage in 1793 CRC patients. (D) The correlation between different cancer stages and SETD2 and SMAD4 mutations in CRC patients was analysed in TCGA database. (E) Survival statistics of patients with neither, SMAD4 not STED2, SETD2 not SMAD4, both mutation in TCGA database.

To investigate whether SETD2 exacerbates SMAD4‐dificiency CRC, we first consulted the clinical data samples of CRC in the TCGA database and analysed the expression levels of SETD2 and SMAD4 in the samples of CRC patients and their relationship with cancer stage and survival rate. The results showed a 5% mutation rate for SETD2 and a 14% mutation rate for SMAD4 in CRC patients (Figure 1A). The TCGA database revealed that a certain correlation was confirmed between SETD2 and SMAD4 mRNA expression levels in CRC patients (Figure 1B). Moreover, the rate of co‐mutation of SETD2 and SMAD4 was significantly increased in patients with advanced CRC (Figures 1C and D), and patients who exhibited co‐mutation of SETD2 and SMAD4 experienced a poorer clinical survival course (Figure 1E). The collective results implied that the deficiency of SETD2 and SMAD4 promotes the progression of CRC to more advanced stages, underscoring the significant roles of SETD2 and SMAD4 in the pathogenesis of CRC.

### 3.2 Loss of SETD2 promotes the formation of intestinal polyposis in S*mad4^Vil‐KO^;Setd2^Vil‐KO^* mice

**FIGURE 2:**
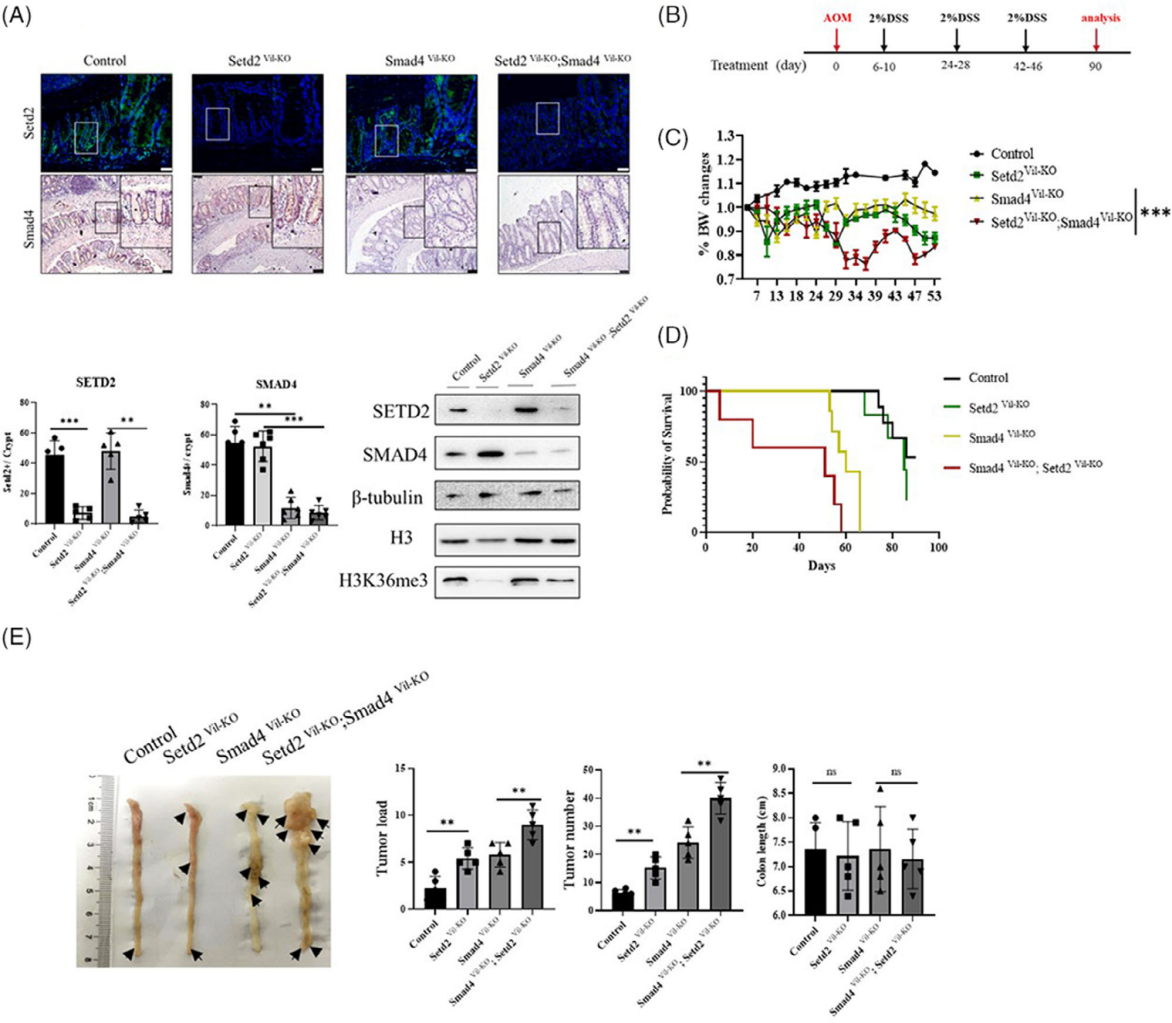
Loss of SETD2 promotes the formation of SMAD4‐deficient CRC in mice. (A) The expressions of SETD2 and SMAD4 were analysed by immunofluorescence and immunohistochemistry, and the expressions of SETD2, SMAD4 and H3K36me3 were analysed by Western blot. Scale bars: 200 μm. Data represent means ± S.D., and statistical significance was determined by a two‐tailed Student *t*‐test. *, *p* < .05; **, *p* < .01; ***, *p* < .001. (B) Schematic representation of the AOM/DSS protocol used to induce CRC in mice. (C and D) Mice were treated with 2% DSS in drinking water after intraperitoneal injection of 10 mg/kg AOM, and losses in body weight (C) and survival (D) were recorded (*n* = 6 per genotype). (E) 90 days after AOM injection, mice were sacrificed to examine tumour burden and colon length (*n* = 6 per genotype). Scale bar: 1 cm.

To assess the role of SETD2 in SMAD4‐mutant CRC, the intestinal epithelium‐specific SETD2 and SMAD4 deficiency mouse strain (*Setd2^F/F^;Smad4^F/F^*; *Villin‐Cre* mice, here we referred as *Smad4^Vil‐KO^;Setd2^Vil‐KO^* mice) were generated by *Villin‐Cre* mice with *Smad4‐flox* ^29^ *;Setd2‐flox* mice (*Smad4^F/F^*;*Setd2^F/F^* mice). As expected, IHC and Western blot (WB) validation confirmed efficient ablation of SETD2 and SMAD4 expression in the intestinal epithelium of *Smad4^Vil‐KO^;Setd2^Vil‐KO^* mice (Figure 2A). CRC was induced with AOM/DSS^30^, ^31^, ^32^ (Figure 2B). During the entire drug feeding period, *Smad4^Vil‐KO^;Setd2^Vil‐KO^* mice exhibited severe reactions, including substantial weight loss, rectal bleeding and obstruction, resulting in significantly lower body weight compared with *Smad4^Vil‐KO^* mice (Figure 2C). All *Smad4^Vil‐KO^;Setd2^Vil‐KO^* mice died within 60 days of drug administration (Figure 2D). The colon lesions of the mice were observed after washing with PBS. Compared with *Smad4^Vil‐KO^* mice, *Smad4^Vil‐KO^;Setd2^Vil‐KO^* mice displayed a higher number of larger polyposis lesions, predominantly located in the colon rather than the rectum. Tumours were indicated with black arrows and no significant difference in colon length between all groups treated with AOM/DSS (Figure 2E). These results collectively demonstrate that the deficiency of SETD2 promotes the development of intestinal lesions in SMAD4‐ablated mice.

### 3.3 The inactivation of SETD2 exacerbates the malignant progression of SMAD4‐deficient CRC in vivo and in vitro

**FIGURE 3:**
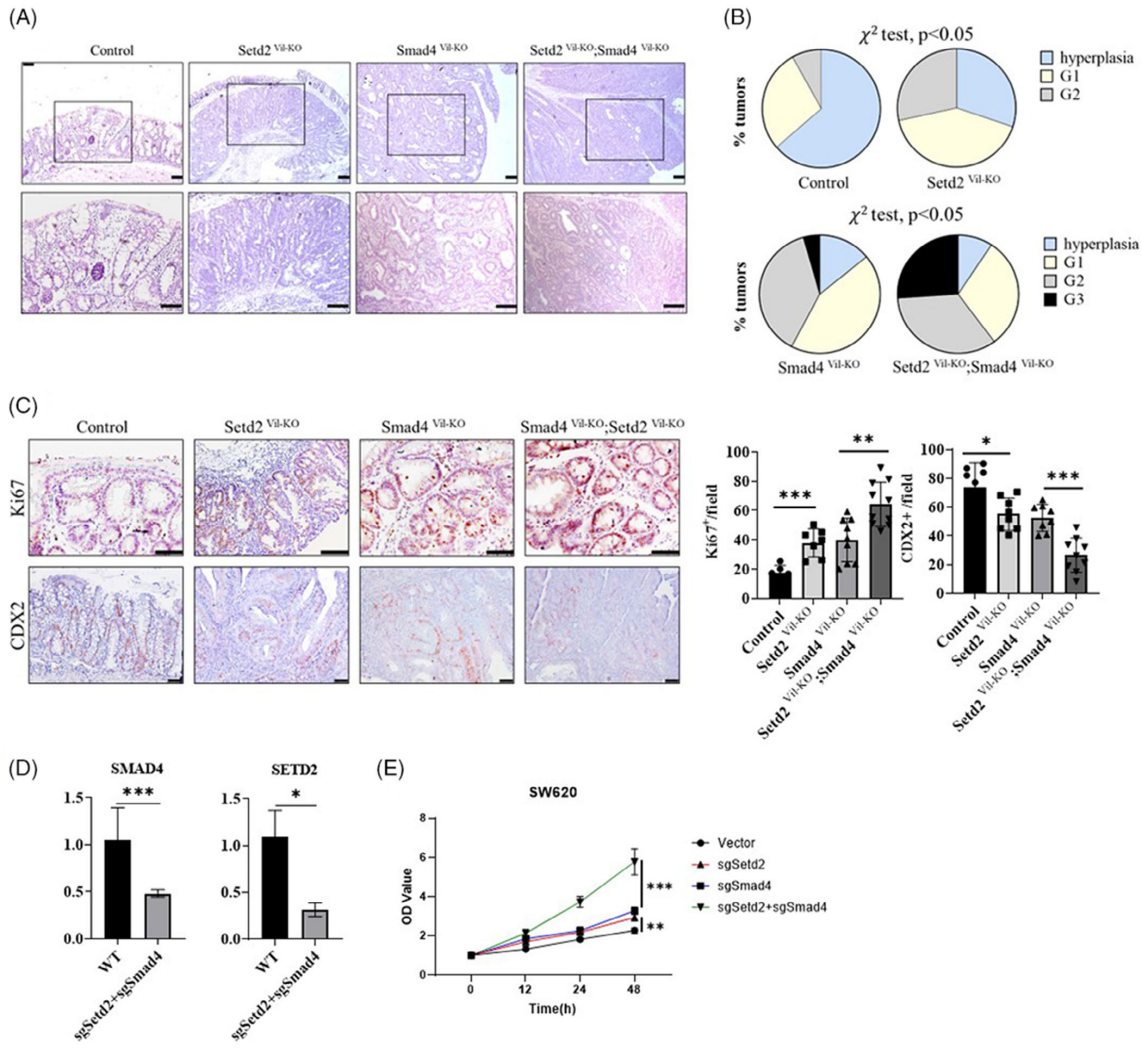
The inactivation of SETD2 exacerbates the malignant progression of SMAD4‐deficient CRC in vivo and in vitro. (A and B) H&E‐stained sections of colon tissue from AOM/DSS‐treated mice, representative images of each genotype colon and overall grade of tumour (*χ*2 test) are shown (*n* = 5 per genotype). Scale bar: upper 200 μm, lower 100 μm. (C) Representative images of Ki67 and CDX2 staining from AOM/DSS‐treated mice of each genotype are shown (*n* = 5 per genotype). Scale bar: 50 μm (top), 100 μm (bottom). Data represent means ± S.D., and statistical significance was determined by a two‐tailed Student *t*‐test. *, *p* < .05; **, *p* < .01; ***, *p* < .001. (D and E) SW620 cells were transfected with empty vector, sgSMAD4 plasmid and sgSETD2 plasmid, and the mRNA expression was detected by real‐time fluorescence quantitative PCR (*n* = 4 per genotype). CCK8 was used for detection. Data represent means ± S.D., and statistical significance was determined by a two‐tailed Student *t*‐test. *, *p* < .05; ***, *p* < .001.

To gain further insights into intestinal lesions in the mouse model, we conducted HE staining on sections of intestinal tissue. Histological staining analysis revealed that tumour formations were more extensive and less differentiated in *Smad4^Vil‐KO^;Setd2^Vil‐KO^* mice (Figure 3A). Histological analysis indicated that approximately 26% of the polyps in the *Smad4^Vil‐KO^;Setd2^Vil‐KO^* mice were classified as poorly differentiated tumours, and poorly and moderately differentiated tumours together accounted for about 60%. In contrast, the tumours in *Smad4^Vil‐KO^* mice were primarily of low grade, with approximately 40% classified as poorly and moderately differentiated tumours (Figure 3B). Ki67 staining showed that the proliferation rate of *Smad4^Vil‐KO^;Setd2^Vil‐KO^* mice is higher than *Smad4^Vil‐KO^* mice (Figure 3C). To further investigate the malignancy of the intestinal tumours, we performed IHC staining for CDX2. CDX2 staining showed a significant decrease in the *Smad4^Vil‐KO^;Setd2^Vil‐KO^* mice (Figure 3C). Therefore, SETD2 ablation aggravates the malignant progression of CRC caused by SMAD4 ablation.

To further explore whether the loss of SETD2 exacerbates the progression of SMAD4‐deficient CRC, we generated CRC cell line SW620 with SMAD4 and SETD2 co‐mutation and observed cell proliferation. The SMAD4^KO^;SETD2^KO^ SW620 cells were validated via qPCR, confirming successful mutations in both genes (Figure 3D). The CCK8 results showed that the proliferation rate of SMAD4^KO^;SETD2^KO^ SW620 cells were significantly higher than SMAD4^KO^ cells (Figure 3E). Through the CCK8 results, we further confirmed that the loss of SETD2 exacerbates the progression of SMAD4‐deficient CRC. Taken together, our results demonstrate that SETD2 deficiency exacerbates the malignant progression of SMAD4‐deficient CRC both in vivo and in vitro.

### 3.4 SETD2 aggravates CRC induced by SMAD4 ablation via activating RAS/ERK signalling pathway

**FIGURE 4:**
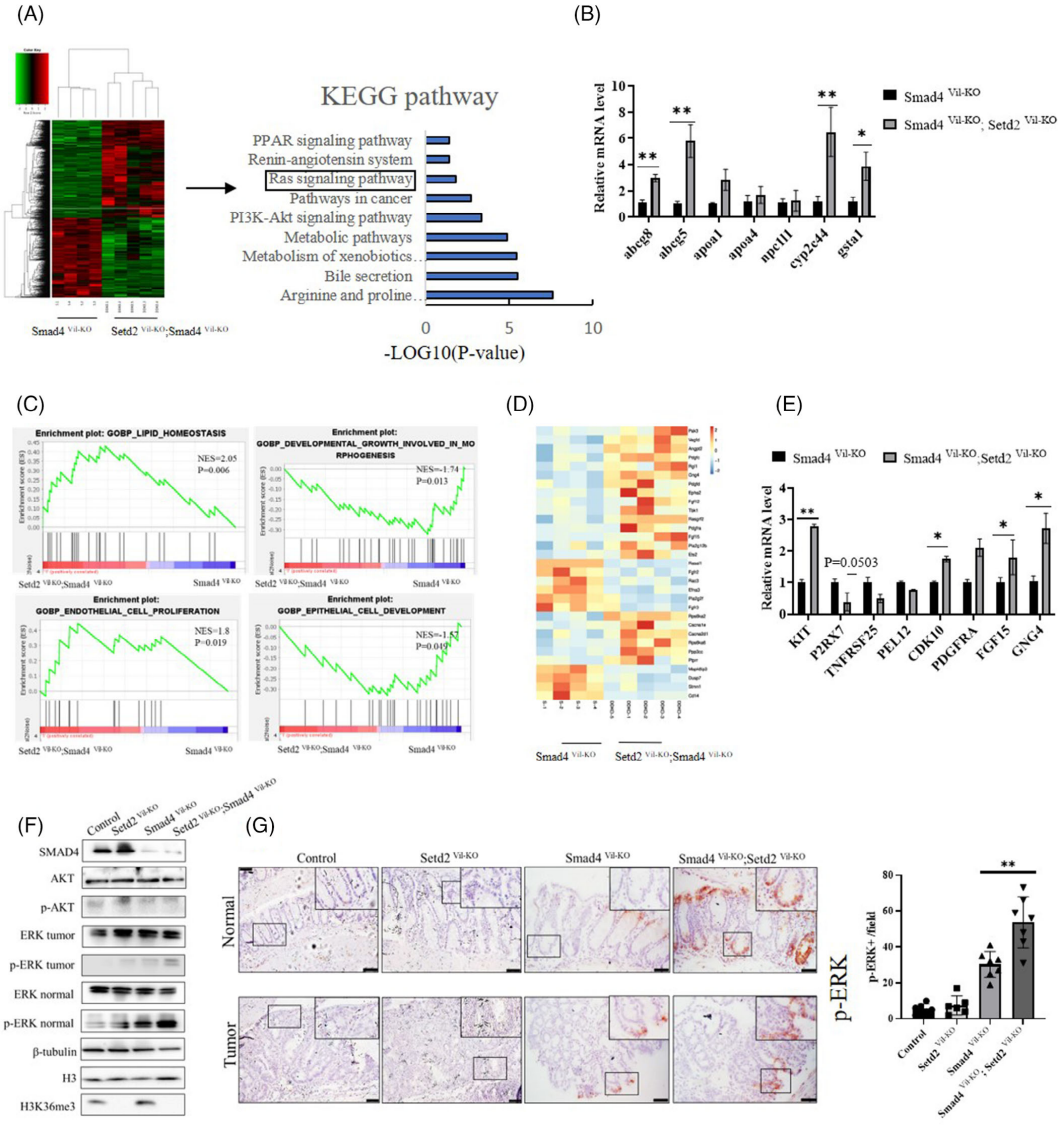
SETD2 aggravates colorectal cancer induced by SMAD4 ablation by activating RAS/ERK signalling pathway. (A) Heat map of RNA‐seq data to compare gene expression in IECs of mice treated with AOM/DSS for 10 d. KEGG term analysis of gene expression changes on the right. (B) mRNA expression levels associated with the Metabolic pathways in IECs of AOM/DSS treated (10 d) mice (*n* = 4 per genotype). (C) GSEA enrichment plots of differentially expressed genes associated with SETD2 deletion. (D) Heatmap summarises the RNA‐seq results of RAS/ERK signalling pathway related gene expression. (E) mRNA expression levels associated with the RAS/ERK signalling pathway in IECs of AOM/DSS‐treated (10 d) mice (*n* = 4 per genotype). (F) Western blot analysis of the indicated proteins in IECs isolated from AOM/DSS‐treated (10 d) mice. (G) p‐p44/p42 MAPK staining in AOM/DSS‐treated (10 d) mice. Scale bar: 100 μm. Data represent means ± S.D., and statistical significance was determined by a two‐tailed Student *t*‐test. **, *p* < .01.

To further explore the mechanism of SETD2 exacerbating CRC caused by SMAD4 deletion, we performed RNA sequencing analysis on IECs of *Smad4^Vil‐KO^* mice and *Smad4^Vil‐KO^;Setd2^Vil‐KO^* mice induced by AOM/DSS for one cycle (Figure 4A). Compared with the *Smad4^Vil‐KO^* mice, RNA sequencing revealed significant changes in the global transcriptome of the *Smad4^Vil‐KO^;Setd2^Vil‐KO^* mice. In total, 975 genes were up‐regulated and 793 were down‐regulated (fold change > 1.5) among all 18 950 genes expressed in *Smad4^Vil‐KO^;Setd2^Vil‐KO^* IECs. KEGG analysis indicated that there was a significant enrichment of genes related to pathways in cancer, metabolism, PI3K‐AKT signalling pathway and RAS signalling pathway (Figure 4A). Notably, several metabolic pathways exhibited substantial activation in the RNA‐seq results (Figure 4A). RT‐qPCR was performed to validate our results (Figure 4B).

Next, we employed gene set enrichment analysis (GSEA) to elucidate SETD2‐mediated signalling. GSEA results indicated changes in metabolic pathways, consistent with the RNA‐seq findings. Additionally, pathways related to endothelial cell proliferation, developmental growth involved in morphogenesis and epithelial cell development were altered in *Smad4^Vil‐KO^;Setd2^Vil‐KO^* IECs (Figure 4C). RNA‐seq also revealed that the genes associated with the MAPK signalling pathway and the RAS signalling pathway were significantly changed (Figure 4D). We confirmed these alterations in mRNA expression levels through RT‐qPCR for genes related to the RAS and MAPK signalling pathways (Figure 4E). We performed WB experiments to measure the protein levels of RAS/ERK signalling pathway (Figure 4F). the WB results revealed that the expression level of p‐ERK was increased in *Smad4^Vil‐KO^* mice compared with wild‐type mice. Moreover, the expression level of p‐ERK was substantially increased in *Smad4^Vil‐KO^;Setd2^Vil‐KO^* mice compared with *Smad4^Vil‐KO^* mice. In addition, the expression level of p‐ERK was also detected by IHC staining (Figure 4G). The IHC staining results were consistent with those of WB. It's important to highlight that our primary focus was on assessing changes in the AKT signalling pathway. However, our WB results (Figure 4F) did not demonstrate a significant up‐regulation of p‐AKT. These results indicated that SETD2 ablation activate the RAS/ERK signalling pathway in *Smad4^Vil‐KO^;Setd2^Vil‐KO^* mice.

### 3.5 SETD2 inhibits CRC development by promoting the transcription of DUSP7 in the RAS/ERK signalling pathway

**FIGURE 5:**
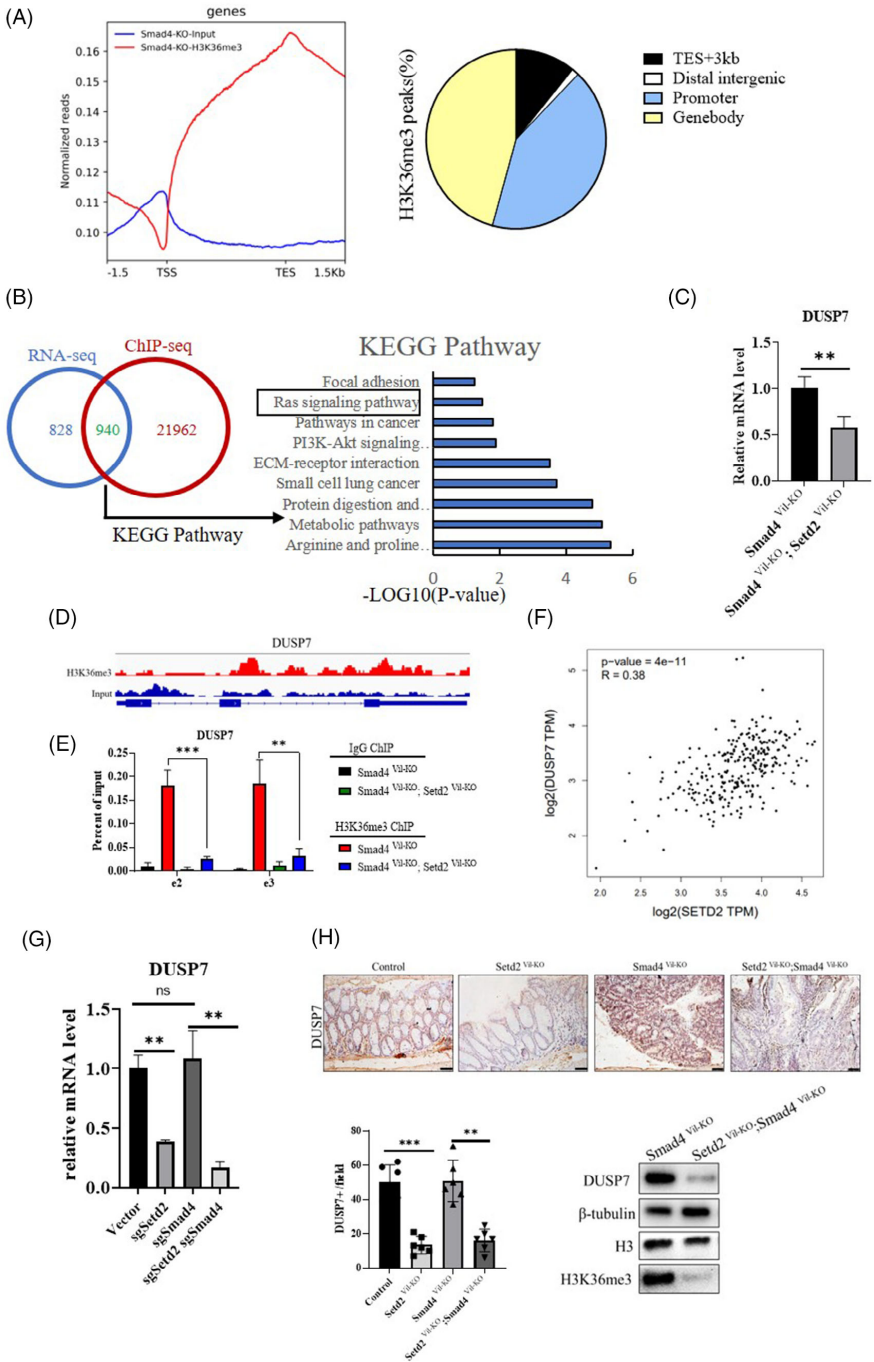
SETD2 inhibits CRC development by promoting the transcription of DUSP7 in the RAS/ERK signalling pathway. (A) Normalised read density of H3K36me3 ChIP‐seq signals in IECs isolated from *Smad4^Vil‐KO^* mice treated for 10 d with AOM/DSS. Analysis of the occupancy of H3K36me3 ChIP‐seq peaks in gene bodies and intergenic regions. (B) Venn diagram showing the number of genes harbouring H3K36me3 binding and displaying expression changes in *Smad4^Vil‐KO^* IECs. Right panel shows the KEGG analysis of the overlapping genes. (C) RT‐ qPCR analysis of DUSP7 expression in *Smad4^Vil‐KO^* and *Smad4^Vil‐KO^;Setd2^Vil‐KO^* mice as indicated. (D) Snapshot of H3K36me3 ChIP‐seq signal at the DUSP7 loci in IECs isolated from AOM/DSS‐treated (10 d) *Smad4^Vil‐KO^* and *Smad4^Vil‐KO^;Setd2^Vil‐KO^* mice. (E) ChIP‐qPCR analysis of H3K36me3 binding for DUSP7 loci in IECs from AOM/DSS‐treated (10 d) *Smad4^Vil‐KO^* and *Smad4^Vil‐KO^;Setd2^Vil‐KO^* mice, and IgG was used as the control (*n* = 3 per genotype). (F) Correlation between SETD2 and DUSP7 expression levels in CRC specimens in TCGA databases. (G) RT‐qPCR analysis of the relative mRNA expression levels in SW620 cells transfected with sgSETD2 and sgSMAD4 plasmids (*n* = 5 per genotype). (H) DUSP7 staining in AOM/DSS‐treated mice. Scale bar: 50 μm. Data represent means ± S.D., and statistical significance was determined by a two‐tailed Student *t*‐test. **, *p* < .01; ***, *p* < .001. Western blot analysis of the DUSP7 protein in IECs isolated from AOM/DSS‐treated mice.

Since SETD2 regulates the expression levels of downstream genes through H3K36me3, loss of SETD2 leads to a decrease in H3K36me3 expression level. We next performed ChIP‐seq analysis on IECs isolated from *Smad4^Vil‐KO^* mice after one cycle of AOM/DSS treatment using H3K36me3 specific antibody to explore the molecular mechanism of SETD2 in SMAD4‐mutated CRC. Totally, 78 769 H3K36me3 peaks were enriched in *Smad4^Vil‐KO^* IECs, of which about 45% were in the gene body region and 42% in the promoter region (Figure 5A). To investigate the relationship between chromatin binding and transcriptional regulation, ChIP‐seq data and expression profiles were integrated for analysis. Including up‐regulated and down‐regulated genes, the Venn diagrams showed that 940 genes had expression changes after SETD2 ablation (Figure 5B). KEGG analysis revealed that these overlapping genes are related to Metabolism, RAS signalling pathway and PI3K‐Akt signalling pathway (Figure 5B). As shown in Figure 4, the expression levels of genes involved in RAS/ERK signalling pathway were down‐regulated after SETD2 mutation, indicating that SETD2‐mediated H3K36me3 may positively regulate certain genes that inhibit RAS/ERK signalling pathway. As expected, the expression level of DUSP7 was down‐regulated in *Smad4^Vil‐KO^;Setd2^Vil‐KO^* mice compared with *Smad4^Vil‐KO^* mice (Figure 5C). DUSP7 (Dual specificity phosphatase 7) is a dual‐specificity phosphatase that possesses phosphatase activity by dephosphorylating specific tyrosine and serine residues of various substrates. Within the RAS/ERK signalling pathway, DUSP7 can dephosphorylate ERK in a site‐specific manner, thereby negatively regulating the activity of ERK protein.^33^, ^34^, ^35^ The intensity of H3K36me3 binding in exon e2 and e3 regions within the DUSP7 gene locus was increased in the H3K36me3 group compared with the Input group in the genome browser tracker (Figure 5D). We further confirmed the presence of H3K36me3 binding in the e2 and e3 regions of DUSP7 through ChIP‐qPCR, and the results indicated that the intensity of H3K36me3 binding at these loci decreased upon SETD2 ablation (Figure 5E). Moreover, based on the PCR data in TCGA database, the mRNA level of SETD2 was significantly positively correlated with DUSP7, which was consistent with our findings (Figure 5F). Consequently, our results suggested that SETD2 ablation led to the inhibition of DUSP7 expression. We performed RT‐qPCR and IHC to verify the expression level of DUSP7 regulated by SETD2 (Figure 5G). SETD2 deficiency down‐regulated the expression level of DUSP7 in both SMAD4 wild‐type and knockout cells. Furthermore, we assessed the expression level of DUSP7 in mice using WB and IHC staining, which revealed that the expression level of DUSP7 was significantly down‐regulated in *Smad4^Vil‐KO^;Setd2^Vil‐KO^* mice compared with *Smad4^Vil‐KO^* mice (Figure 5H). Taken together, our results indicate that SETD2 regulates DUSP7 to suppress the activation of RAS/ERK signalling pathway and inhibit the progression of SMAD4‐deficient CRC.

### 3.6 Inhibition of the RAS/ERK signalling pathway suppressed the cancer progression

**FIGURE 6:**
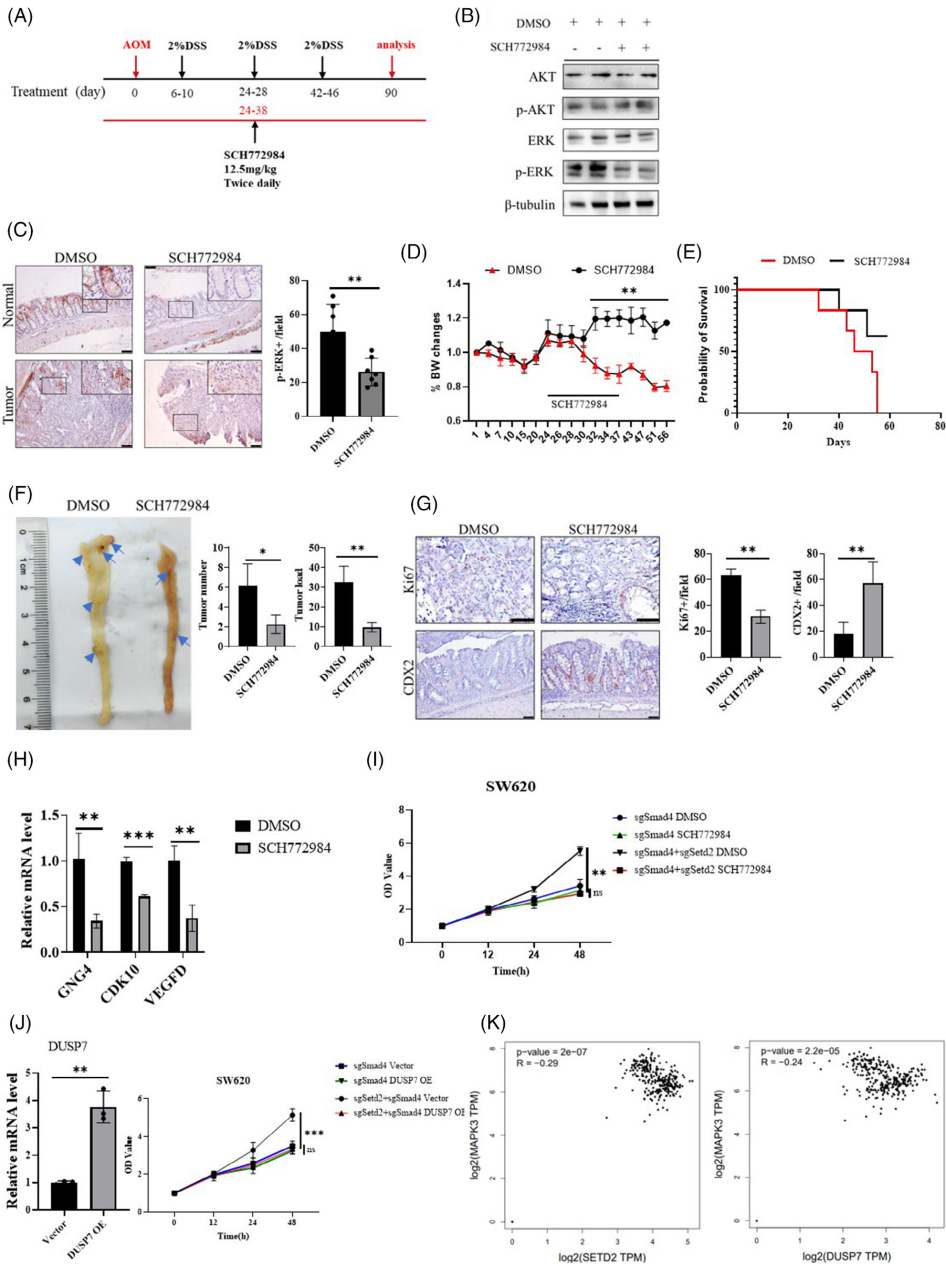
Inhibition of the RAS/ERK signalling pathway alleviated the cancer progression. (A) Schematic representation of the SCH772984 protocol used to treat CRC in mice. (B) Western blot analysis of the indicated proteins in IECs isolated from DMSO and SCH772984‐treated mice. (C) p‐p44/p42 MAPK staining in DMSO and SCH772984‐treated mice. Scale bar: 100 μm. Data represent means ± S.D., and statistical significance was determined by a two‐tailed Student *t*‐test. **, *p* < .01. (D and E) Mice were treated with DMSO and SCH772984, and losses in body weight (D) and survival (E) were recorded (*n* = 6 per genotype). (F) 90 days after AOM injection, mice were sacrificed to examine tumour burden (*n* = 6 per genotype). Scale bar: 1 cm. (G) Representative images of Ki67 and CDX2 staining are shown in tumours from mice treated with DMSO and SCH772984 after AOM/DSS‐induced CRC (*n* = 5 per genotype). Scale bar: 50 μm (top), 100 μm (bottom). Data represent means ± S.D., and statistical significance was determined by a two‐tailed Student *t*‐test. *, *p* < .05; **, *p* < .01; ***, *p* < .001. (H) mRNA expression levels associated with the RAS/ERK signalling pathway in IECs of DMSO and SCH772984‐treated mice (*n* = 4 per genotype). (I) the SMAD4^KO^ and SMAD4^KO^;SETD2^KO^ cells were treated by DMSO and SCH772984, respectively (*n* = 4 per genotype). CCK8 was used for detection. Data represent means ± S.D., and statistical significance was determined by a two‐tailed Student *t*‐test. **, *p* < .01. (J) RT‐ qPCR analysis of DUSP7 expression in SW620 transfected with vector and DUSP7 OE plasmids, respectively (*n* = 3 per genotype). CCK8 was used for detection. Data represent means ± S.D., and statistical significance was determined by a two‐tailed Student *t*‐test. **, *p* < .01; ***, *p* < .001. (K) Correlation between SETD2/ DUSP7 and MAPK3 expression levels in CRC specimens in TCGA databases.

To further validate the relationship between SETD2 and the RAS/ERK signalling pathway in SMAD4‐deficient CRC, the ERK inhibitor SCH772984^36^ was administered to *Smad4^Vil‐KO^;Setd2^Vil‐KO^* mice as a therapeutic intervention. After the end of the first cycle of DSS administration, SCH772984 was administered to the mice via intraperitoneal injection twice daily for 14 consecutive days^37^, ^38^ (Figure 6A). IHC and WB experiments revealed a significant decrease in p‐ERK levels in colon tissues (Figures 6B and C). Throughout the treatment period, the mice in the inhibitor group displayed notably higher body weights and survival rates compared with those in the control group (Figures 6D and E). After AOM/DSS induction, the mice were then sacrificed to remove the colon. The number and size of tumours in treatment group were significantly lower than those in the control group (Figure 6F). Furthermore, IHC staining results for Ki67 and CDX2 revealed that the proliferation rate of treatment group was inhibited and the CDX2 expression was increased in the treatment group. These results suggested that SCH772984 effectively reduced the malignancy of the cancer (Figure 6G). Additionally, the mRNA levels of downstream genes in the RAS/ERK signalling pathway were significantly decreased (Figure 6H). Moreover, to further explore the link between SETD2 and RAS/ERK signalling pathway, the ERK inhibitor SCH772984 was applied on SMAD4^KO^ and SETD2^KO^;SMAD4^KO^ SW620 cells, respectively (Figure 6I). The proliferation of SMAD4^KO^ cells was not affected by SCH772984, while the proliferation of SETD2^KO^;SMAD4^KO^ cells were significantly inhibited. In addition, DUSP7 OE plasmid was also transfected in SETD2^KO^;SMAD4^KO^ SW620 cells (Figure 6J). We confirmed that DUSP7 was expressed by RT‐qPCR, and the data showed that the overexpression of DUSP7 significantly inhibited the cell proliferation in SETD2^KO^;SMAD4^KO^ SW620 cells. Finally, we further verify the clinical relevance between SETD2/DUSP7 and RAS/ERK signalling in the TCGA database. Consistent with our study, there were significantly negative correlations between expression level of MAPK3 and that of SETD2 or DUSP7, respectively (Figure 6K). All of these works demonstrate that inhibiting the RAS/ERK pathway in vivo and in vitro can alleviate CRC caused by SETD2 deficiency.

## 4 DISCUSSION

CRC is a widespread global health concern, ranking third in incidence among both sexes, posing a significant threat to human lives.^39^ In our study, the high frequency of SETD2 and SMAD4 mutations in advanced CRC and the poor survival of patients with SETD2 and SMAD4 mutations confirmed the important roles of SETD2 and SMAD4 in CRC. Our results showed that *Smad4^Vil‐KO^;Setd2^Vil‐KO^* mice developed a more severe CRC phenotype. Activation of the RAS/ERK pathway was found by RNA‐seq, which was further confirmed by IHC and WB. Next, ChIP‐seq was performed to identify the molecular targets of SETD2. By combining RNA‐seq and ChIP‐seq data, DUSP7 was identified as a significantly altered target. Subsequent IHC and WB analyses revealed that SETD2 mutation led to the down‐regulation of DUSP7 expression, subsequently activating the RAS/ERK signalling pathway. Finally, we treated cancer‐induced *Smad4^Vil‐KO^;Setd2^Vil‐KO^* mice with the ERK inhibitor SCH772984 and found that the inhibitor effectively inhibited the development of CRC. Additionally, the overexpression of DUSP7 in SETD2^KO^;SMAD4^KO^ SW620 cells were also inhibited the proliferation of cells.

The SMAD family is a crucial intracellular signalling component within the TGF‐β signalling pathway, with SMAD4 serving as the central signal transduction molecule.^7^ TGF‐β signalling is mediated through two TGF‐β receptors situated on the cell membrane: the TGF‐β type I receptor and TGF‐β type II receptor (TGFBR1, TGFBR2).^40^, ^41^ Upon ligand binding, these receptors phosphorylate downstream R‐SMAD proteins, activating their transcriptional regulatory functions. As a downstream transcriptional regulator, R‐SMAD binds to Co‐SMAD (SMAD4) and translocates to the nucleus to exert its transcriptional regulatory activity.^42^, ^43^ SMAD4 has a 14% mutation rate in CRC, indicating its important role in CRC. Deletion of SMAD4 in CRC represents a distinct CRC subtype. Smad4‐deficient CRC patients had a shorter recurrence‐free survival. In embryos, deletion of SMAD4 blocked gastrulation.^29^ Mutations in SMAD4 lead to the formation of gastrointestinal tumours.^9^ Deletion of SMAD4 promotes the up‐regulation of NLE1, which leads to the growth and metastasis of CRC.^44^ In addition, SMAD4 also plays an important role in PDAC and lung cancer.^45^, ^46^

Histone modifications are one of the important forms of epigenetic regulation, of which histone methylation is a key determinant of chromatin status.^47^ SETD2 is the only known H3K36 trimethyltransferase. It has been reported that SETD2 plays an important role in cancer. Notably, SETD2 deficiency causes experimental colitis^15^ and in clinical data, we have observed a tendency for SETD2 deficiency to be associated with CRC. In this study, we found that the simultaneous deletion of SETD2 and SMAD4 in mice led to an increased number and size of colorectal tumours and reduced survival rates, aligning with clinical data. Our experimental results indicated that SETD2 deletion alone did not lead to ERK activation, and SMAD4 deletion alone only leads to partial activation of ERK. However, the co‐mutation of SETD2 and SMAD4 activated the RAS/ERK signalling pathway, suggesting that SETD2 and SMAD4 have synergistic effects in suppressing the development of CRC.

The RAS/ERK pathway is activated by most growth factors, cytokines and immune receptors, integrin and chemokine receptors. Receptor tyrosine kinase binds to extracellular growth factors and activates RAS, initiating downstream intracellular signalling.^48^ SETD2 directly affects the transcriptional initiation of Fgfr3 through H3K36me3 modification in the distal promoter region, thereby activating ERK signalling pathway to regulate endoderm differentiation.^21^ In CRC, mutations in a variety of genes can activate ERK, such as METTL3^49^, EGFR^50^ and BRAF.^51^ There is also evidence that SMAD4 deficiency prolongs TGF‐β‐mediated ERK phosphorylation and activation in HCT116 cells.^52^ In our study, SMAD4 deficiency led to up‐regulation of ERK, and SETD2 deficiency resulted in down‐regulation of DUSP7, which enhanced ERK activity. The DUSP7, located on human chromosome 3p21, which specifically binds to p‐ERK1/2 and dephosphorylates it to inhibit the MAPK signalling pathway.^53^, ^54^ DUSP7 is associated with a set of cellular activities, including promoting the meiosis of oocytes,^55^ promoting T cell differentiation,^56^ and losing the versatility of embryonic stem cells.^57^ Notably, the loss of DUSP7 has been linked to chromosomal fusion defects.^58^ DUSP7 plays an important role not only in development, but also in tumours. Loss of DUSP7 promotes oestrogen‐dependent growth of breast cancer cells,^59^ and increased DUSP7 expression levels have been associated with reduced proliferation and invasion of renal cancer cells.^60^ However, the relationship between DUSP7 and tumourigenesis of CRC has not been reported. In our work, we found that the expression level of DUSP7 is regulated by SETD2. Deletion of SETD2 led to a decrease of DUSP7 expression, resulting in the up‐regulation of ERK activity and the activation of the RAS/ERK signalling pathway. It has been reported that the co‐mutation of RAS, SMAD4 and TP53 in metastatic CRC is associated with poor prognosis while the mutation of RAS or TP53 alone is not,^61^ which reflected the complexity of CRC development. CRC development is often associated with multiple genetic mutations. It has been reported that the co‐mutation of SMAD4 with TP53 or Catnb leads to malignant development of CRC.^62^, ^63^ In our study, we found that SETD2 deficiency exacerbated the malignant development of SMAD4‐deficient CRC through the RAS/ERK signalling pathway. This study indicates that SETD2 might be an upstream regulator of the RAS/ERK signalling pathway. Moreover, whether SETD2 deletion enhanced the metastasis of SMAD4‐deficient CRC needs to be further studied.

## SIGNIFICANCE

SETD2‐mediated H3K36me3 inhibits the RAS/ERK signaling pathway by facilitating the transcription of DUSP7 in SMAD4‐deficient CRC, which could provide a potential therapeutic target for the treatment of advanced CRC.

## CONFLICT OF INTEREST STATEMENT

The authors have declared that no conflict of interest exists.

## Supporting information

Supporting Information

## Data Availability

All data are available from the authors upon reasonable request. RNA‐seq and ChIP‐seq raw data have been deposited in the Gene Expression Omnibus (GEO) under accession number GEO: GSE 228023 and GSE 228024.
